# Acquired von Willebrand Syndrome Secondary to Normally Functioning Mechanical Aortic Valve and High-Output Cardiac State

**DOI:** 10.3390/jcdd9120454

**Published:** 2022-12-12

**Authors:** Xinglin Yang, Jinghong Zhang, Yamin Lai, Xuemin Yan, Xiaoxiao Guo, Jianhua Han, Jihai Liu, Jiangshan Wang, Huadong Zhu

**Affiliations:** 1 Department of Cardiology, Peking Union Medical College Hospital, Chinese Academy of Medical Sciences & Peking Union Medical College, Beijing 100730, China; 2 Central Clinical School, Monash University, Melbourne, VIC 3004, Australia; 3 Department of Gastroenterology, Peking Union Medical College Hospital, Chinese Academy of Medical Sciences & Peking Union Medical College, Beijing 100730, China; 4 Department of Clinical Laboratory, Peking Union Medical College Hospital, Chinese Academy of Medical Sciences & Peking Union Medical College, Beijing 100730, China; 5 Emergency Department, State Key Laboratory of Complex Severe and Rare Diseases, Peking Union Medical College Hospital, Chinese Academy of Medical Sciences & Peking Union Medical College, Beijing 100730, China

**Keywords:** von Willebrand factor, gastrointestinal bleeding, anemia, heart valve disease

## Abstract

Acquired von Willebrand syndrome (AVWS) is caused by an acquired deficiency of von Willebrand factor (VWF), a multimeric protein required for primary hemostasis. For patients with heart valve diseases, high gradient across the malfunctioning valves could cause elevated shear stress and damage the most effective large VWF, eventually resulting in AVWS. However, AVWS has not been reported in association with normally functioning mechanical valves. Herein, we reported a 74-year-old female who suffered from recurrent gastrointestinal bleeding with a history of mechanical aortic and mitral valve replacement. This patient’s function/antigen ratio of VWF was decreased and gel electrophoresis revealed the loss of large VWF, which confirmed the diagnosis of AVWS. Echocardiogram showed that the function of the prostheses was normal. However, the gradient across aortic valve was increased due to a high cardiac state which is secondary to chronic anemia, resulting in the disruption of large VWF multimers and exacerbation of gastrointestinal (GI) bleeding. After managing the patient’s anemia with transfusion, the gradient across the aortic valve had improved, with the resolution of GI bleeding. This is the first case report of AVWS that is associated with a normally functioning mechanical valve. AVWS should be considered one of the differential diagnoses if patients present with unexplained GI bleeding on the background of having prosthetic heart valves. The management of the underlying condition is essential.

## 1. Introduction

Von Willebrand disease (VWD) is characterized by quantitative or qualitative abnormalities of von Willebrand factor (VWF), which is a crucial protein for hemostasis. Heritable VWD, which is caused by pathogenic variants in the *VWF* gene, is the most common inherited bleeding disorder. On the other hand, acquired von Willebrand syndrome (AVWS) is much less common and is related to several conditions that affect VWF levels and activity. AVWS is often under-diagnosed [1].

VWF is a large glycoprotein which is synthesized and released by megakaryocytes and endothelial cells. It can be cleaved to a spectrum of multimer sizes. VWF functions in primary hemostasis by forming an adhesive bridge between platelets and vascular subendothelial structures, as well as between adjacent platelets. High-molecular-weight multimers are the most active forms of VWF [2].

Mechanisms of reduced VWF in AVWS involve reduced synthesis, antibody-mediated destruction, adsorption onto cells or proteins, and shear stress-induced proteolysis [1]. AVWS can occur secondary to heart valve diseases (HVD) with high shear stress. Under high-shear conditions, large VWF multimers can change to an elongated form and be cleaved by an enzyme (ADAMTS13), converting into smaller and less efficient multimers. The most widely known HVD-associated AVWS is Heyde syndrome—a condition of gastrointestinal bleeding (GI) from angiodysplasia in the presence of aortic stenosis (AS). In addition to AS, aortic or mitral regurgitation and dysfunction of prosthetic valves have also been identified as the underlying causes of AVWS [3,4,5,6].

However, AVWS has not been found to be associated with properly functioning mechanical valves. Herein, we reported a case of a 74-year-old female who initially presented with GI bleeding and was subsequently diagnosed with AVWS.

## 2. Case Report

A 74-year-old female presented to the emergency department of Peking Union Medical College Hospital with a 1-week history of melena. The patient also complained of fatigue and light-headedness. In the last three years, the patient had been admitted repeatedly to other hospitals for the management of GI bleeding and anemia. The patient received blood transfusions several times. However, the patient’s hemoglobin level was never corrected back to the normal range (110–150 g/L) and they were discharged from the other hospitals when their symptoms were resolved after transfusion.

In terms of past medical history, the patient was diagnosed with rheumatic heart disease and underwent surgical valve replacement of the aortic and mitral valves for stenosis in 2000. The surgery was uneventful. A 21 mm St. Jude aortic prosthesis and a 27 mm St. Jude mitral prosthesis were implanted. The patient had been taking warfarin 3 mg once daily post-surgery and had no family history of bleeding.

On examination, patient was alert and oriented. Vital signs were normal (temperature 36.5 °C, blood pressure 127/62 mmHg, heart rate 73/min, breathing rate 20/min, oxygen saturation 98% on room air). Cardiac auscultation revealed opening and closing sounds of mechanical valves.

Blood test showed that hemoglobin was 49 g/L (110–150 g/L) and mean corpuscular volume was 85.8 fL (82.0–97.0 fL); platelet count was 218 × 10^9^/L (100–350 × 10^9^/L) and white blood cell count was 6.8 × 10^9^/L (3.5–9.5 × 10^9^/L). Prothrombin time was 12.4 s (10.4–12.6 s) and activated partial thromboplastin time was 23.3 s (23.3–32.5). Liver and kidney function was normal.

Transthoracic echocardiogram (TTE) showed that the leaflet motions of the prosthetic valves were normal and found no regurgitation or para-valvular leakage. The mitral peak E wave velocity (1.8 m/s) and the mean mitral transvalvular gradient (5 mmHg) were both within the normal range (<1.9 m/s and ≤5 mmHg, respectively). However, the peak velocity across the aortic prosthesis was 3.1 m/s and the mean transvalvular gradient was 22 mmHg, both of which were higher than the normal range (<3 m/s and <20 mmHg, respectively). Her Doppler velocity index (DVI) was 0.38 and the acceleration time (AT) was 85 ms, which were incompatible with prosthetic aortic stenosis (<0.3 and <100 ms, respectively). The effective orifice area indexed (EOAI) was 1 cm^2^/m^2^, not in conformity with patient-prosthetic mismatch (<0.8 cm^2^/m^2^). Hence, the higher-than-expected gradient was due to a high-output cardiac state.

The bleeding source was not identified with upper endoscopy or colonoscopy. Wireless video capsule endoscopy revealed dilated, tortuous submucosal veins, as well as red patches in the small intestine, which were consistent with findings of angiodysplasia.

**Figure 1:**
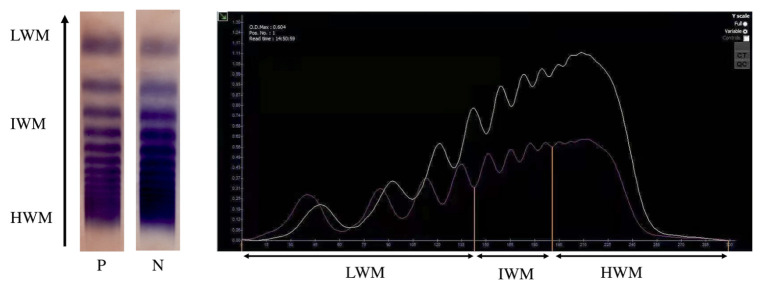
Multimer analysis of von Willebrand factor using agarose gel electrophoresis and densitometry patterns from the test system (Sebia, France) showing the loss of high and intermediate molecular weight von Willebrand factor. (P = this patient, N = control, LMW = low molecular weight, IMW = intermediate molecular weight, HMW = high molecular weight).

Considering the elevated gradients across the aortic valve, AVWS was suspected. Screening tests showed that the VWF antigen (VWF:Ag) was 96.6 IU/dL, which was normal (>50 IU/dL). However, the VWF functional assay (VWF:Act) was below the normal range (>50 IU/dL), standing at 47.7 IU/dL. The ratio of VWF:Act to VWF:Ag was 0.49. Agarose gel electrophoresis revealed loss of high and intermediate molecular weight (HMW/IMW) VWF (Figure 1).

Laboratory tests to screen for other underlying causes of AVWS were performed. Peripheral blood smear found no blasts or atypical cells. Serum protein electrophoresis did not show M-spike. Anti-nuclear antibodies were positive at a low titer (1:80) with speckled fluorescence. Antibodies to double-stranded deoxyribonucleic acid and extractable nuclear antigens were negative. Serum triiodothyronine, thyroxine, and thyroid-stimulating hormone concentration were normal.

The patient was diagnosed with AVWS. The management principle of bleeding is to restore the level of VWF in the circulation and treat the underlying causes by offering VWF concentrates. However, VWF concentrates were not available in the pharmacy of hospitals in mainland China. Given the high risk of thrombosis, desmopressin and recombinant activated factor VII were not considered for this patient. Conservative management was initiated and red blood cell transfusion was administered. As a result of the initial management, the patient’s anemia improved and the GI bleeding stopped. When the patient’s hemoglobin count went back to 118 g/L, TTE showed that the previously elevated peak velocity and mean gradient across the aortic prosthesis had returned to baseline (2.4 m/s and 12 mmHg, respectively). Tests of VWF were repeated and the results demonstrated that the VWF:Act had returned to the normal range (146.3 IU/dL) and the large VWF multimers were completely resolved.

Considering the high thromboembolic risk for this patient with valvular prosthesis, warfarin was re-started two weeks post-discharge with a targeted INR at 1.5–2.0. Duration of the discontinuation was about one month. In terms of follow-up, the patient did not report any recurrence of GI bleeding for a total duration of eight months, with her hemoglobin level being consistently above 100 g/L.

## 3. Discussion

In 1958, Heyde first described the association between AS and GI bleeding [7]. The combination of AS and GI bleeding was therefore termed Heyde syndrome, originating from AVWS induced by high shear stress around the stenotic aortic valve [8]. Subsequently, numerous observations have shown intimate relationships between AVWS and other forms of HVD, including native regurgitant HVD and deterioration of prosthetic valves. Nevertheless, this is the first case report of AVWS that is associated with a well-functioning mechanical valve. Capsule endoscopy revealed that the patient had small bowel angiodysplasia (SBA). Meanwhile, the patient was on continuous anticoagulant medication, which was an important risk factor for GI bleeding in patients with SBA. SBA may have initiated GI bleeding and anemia. The patient’s transvalvular gradient of the prosthetic aortic valve increased due to a high-output cardiac state, which was a physiological response to anemia. As a result, the size and shape of large VWF multimers were disrupted. Once the patient’s hemoglobin count was corrected back to the normal range, the valvular gradient was normalized, as well as the restoration of large VWF multimers.

VWD can be classified into six different types. Type 1 and Type 3 represent quantitative deficiency (partial or complete, respectively). Type 2 comprises four subtypes: (i) 2A, reflecting a loss of HMW VWF (and usually also IMW VWF); (ii) 2B, reflecting a hyper-adhesive VWF, leading to clearance of VWF from the circulation; (iii) 2N, reflecting a loss of factor VIII binding function; and (iv) 2M, reflecting other qualitative VWF defects [9]. AVWS-related high shear stress has laboratory and phenotypic features of type 2A VWD [10]. For initial testing, VWF:Ag and VWF:Act are recommended [11]. They are quantitative measurements and functional assays of VWF, respectively. VWF:Ag or VWF:Act < 50 IU/dL in a patient with a bleeding history confirms the diagnosis. The function/antigen ratio (VWF:Act/Ag) is used to identify reduction in HMW VWF multimers [12]. A ratio of <0.7 is considered discordant [13]. In this case, the VWF:Act was 47.7 IU/dL, and VWF:Act/Ag was 0.49. Analysis using agarose gel electrophoresis confirmed the loss of HMW and IMW VWF. As the patient underwent an uneventful heart surgery and had no family history of bleeding, congenital VWD was excluded and the diagnosis of AVWS was hence established.

It is reported that the rate of AVWS is 67% to 92% among patients with severe AS [14]. The valvular gradient has been shown to be correlated with the degree of disturbance in multimer patterns and the likelihood of bleeding [15]. Aortic and mitral regurgitation were also reported to be correlated with AVWS [4,6]. With regards to prosthetic valves, Blackshear et al. reported abnormal VWF pathway in 43 patients with a dysfunctional aortic or mitral prosthesis [6]. Pérez-Rodríguez et al. also documented acquired VWF qualitative alterations in mitral valve prosthesis leakage [16]. To our knowledge, no data has been reported on patients with normally functioning prosthetic valves. It is important to note that all prosthetic valves are inherently mildly stenotic, giving rise to the potential of increased valvular gradient [17,18]. Furthermore, a high-output cardiac state could further elevate this gradient. In this case, despite the normal structure and motion of the aortic valve, the transvalvular gradient was increased. The American Society of Echocardiography algorithm for prosthetic aortic stenosis was used for further evaluation [18], which supported that the elevated gradient was due to a high-output cardiac state (DVI > 0.3, AT < 100 ms and EOAI > 0.8 cm^2^/m^2^). In fact, once the anemia was properly treated and the cardiac output improved, the gradient was normalized.

In addition to cardiovascular and valvular diseases, etiology of AVWS also includes lymphoproliferative disorders, myeloproliferative neoplasms, autoimmune disorders, and hypothyroidism [19]. Our patient did not have any symptoms relevant to these conditions, and laboratory tests found no evidence to support the diagnosis of such diseases.

Overall, this is the first case report of AVWS in a patient with a normally functioning mechanical valve, which is associated with an increased aortic transvalvular gradient secondary to high-output cardiac state. A vicious cycle formed between anemia, high-output cardiac state, increased pressure gradient across the mechanical valve, build-up of shear stress, loss of large VWF multimers, and GI bleeding. After managing patient’s anemia with transfusion, their hemoglobin level was restored back to normal, along with normalization of the transvalvular gradient and cessation of GI bleeding. In conclusion, if a bleeding patient has a prosthetic valve, AVWS should be taken into consideration even if the function of the prosthesis is normal. The treatment of the underlying conditions is essential for this group of patients.

## Data Availability

The data that support the findings of this study are available from the corresponding author upon reasonable request.
